# Supplementary material for: Enhanced interfacial water dissociation on a hydrated iron porphyrin single-atom catalyst in graphene
Source: Commun Chem. 2023 Nov 2;6:236. doi: 10.1038/s42004-023-01027-9 (PMC10622426; doi:10.1038/s42004-023-01027-9)
Supplement: Supplementary file 2 — Supplementary Information [file 42004_2023_1027_MOESM2_ESM.pdf]

Supplementary Information for
Enhanced interfacial water dissociation on a hydrated iron porphyrin single-atom catalyst in graphene

Laura Scalfi,† Maximilian R. Becker,† Roland R. Netz,† and Marie-Laure Bocquet*,‡

†Fachbereich Physik, Freie Universität Berlin, Arnimallee 14, 14195 Berlin, Germany

‡Laboratoire de Physique de l'École Normale Supérieure, ENS, Université PSL, CNRS, Sorbonne Université, Université Paris Cité, F-75005 Paris, France

E-mail: marie-laure.bocquet@ens.fr

Supplementary Methods

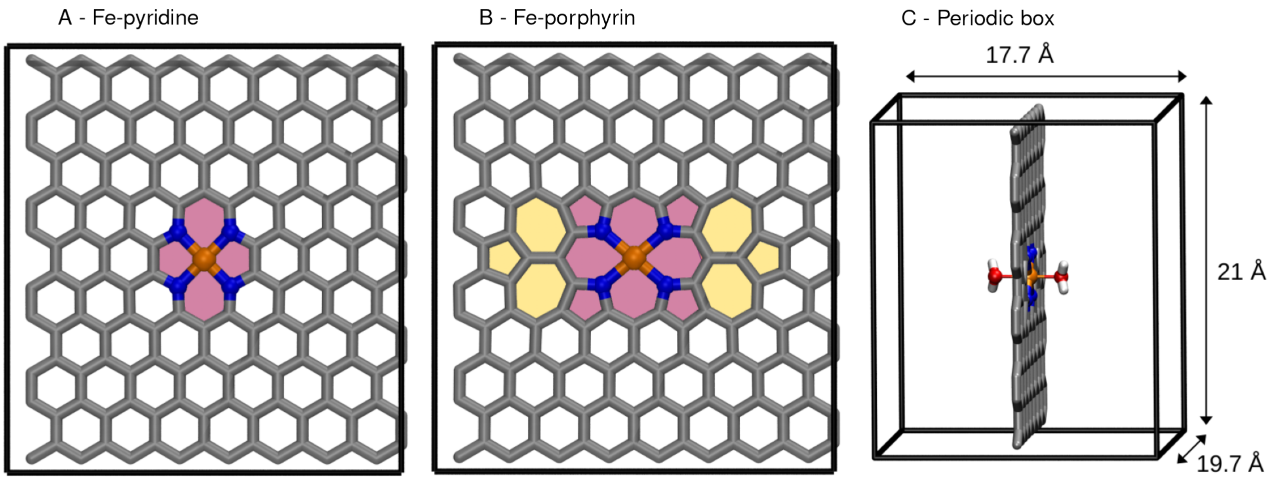

Supplementary Figure 1: Top views of the simulated defects: Fe-pyridine (A) and Fe-porphyrin (B). The periodic box boundaries are drawn in black. (C) Side view of the periodic boxes.

In this study, we consider two different substrates, composed of one iron (Fe) atom coordinated by 4 nitrogens embedded in a single graphene sheet. We choose the previously studied Fe-pyridine defect (with 1 Fe, 4 N and 154 C atoms) and created a Fe-porphyrin defect (with 1 Fe, 4 N and 151 C atoms). The geometry of the two defects is optimized with density functional theory (DFT) to obtain the lateral dimensions of the simulated box: 19.6896×21.3146 Å^2 for the Fe-pyridine defect and 19.6907×21.0636 Å^2 for the Fe-porphyrin defect. The

optimized simulation cell is shown in Supplementary Figure 1A-B for both defects. The distribution of the C-C bond lengths for both periodic Fe-SAC defects is shown in Supplementary Figure 2. The Fe-pyridine only shows slight deviations from the average C-C distance in pure graphene. For Fe-porphyrin, larger deviations corresponding to the five-member rings are present.

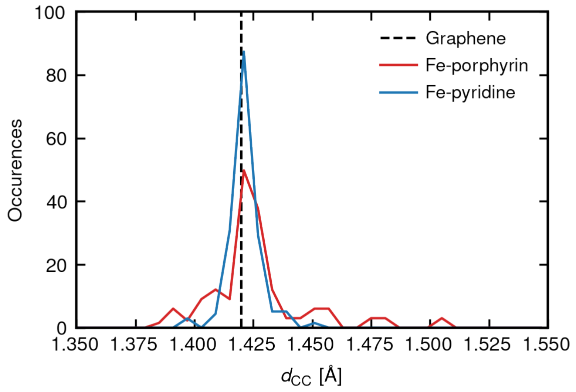

Supplementary Figure 2: C-C distance d_{CC} distribution for Fe-porphyrin (red line) and Fe-pyridine (blue line). The dashed vertical line indicates the C-C distance in graphene.

Additionally, we perform geometry optimizations of Fe-SAC defects with either two adsorbed water molecules or one water molecule and one hydroxide ion adsorbed to the Fe ion (see Figure 1F-G of the main text and Supplementary Figure 1C). Three initial configurations are considered for each system, with the dipole moment of the adsorbed molecules either parallel or perpendicular to the surface. Adsorption energies ΔE_{ads} are calculated including the counterpoise correction to correct for the basis set superposition error (BSSE) using the implementation of CP2K.

The Fe-SAC defects are then solvated by 201 water molecules and equilibrated in the NPT ensemble with the SPC/E water model[1] and the GROMOS 53A6 classical force field[2] for the substrate-water interaction, using the GROMACS simulation package.[3] This leads to a periodic box length of 17.7206 Å in the direction perpendicular to the defects. A side view of the periodic box is shown in Supplementary Figure 1C (shown only with two adsorbed water molecules). Deprotonated configurations are generated by manually deleting a hydrogen bonded to an adsorbed oxygen atom and placing it on a water molecule as far away as possible in the periodic box from the created hydroxide ion. All MD simulations are then run with a CSVR thermostat at 323.15 K, with a timestep of 0.5 fs for at least 10 ps. Details of the simulations are given in Table III of the main text and in Supplementary Table 1. If not stated otherwise, DFT calculations (static and MD) are performed with the software CP2K 7.1,[4] using the PBE-D3 functional[5,6] with Grimme's dispersion correction[7] and the DZVP-MOLOPT-SR basis set,[8] combined with GTH pseudopotentials,[9] and with a cutoff of 600 Ry.

Spin settings: We run both restricted and unrestricted Kohn-Sham simulations (RKS and UKS respectively). UKS DFT simulations solve the Kohn-Sham equations distinguishing spin-up and spin-down electrons, allowing to have non zero spins in the material, while RKS simulations consider only doubly occupied orbitals. In particular, we use the UKS DFT implementation using the orbital transformation solver (OT) by fixing the spin multiplicity of the total system $M = 2S + 1$, with S the spin value of the total system. This implementation of UKS does not allow

to fix the spin on a given atom nor to have a fluctuating total multiplicity during the simulation, but allows for the spin to localize on given atoms (here the Fe atom) and to fluctuate in time. In this work, we perform UKS DFT simulations at two fixed multiplicities M: either $M = 1$ ($S = 0$) or $M = 3$ ($S = 1$). We calculate the spin polarization on the Fe atom s_{Fe} as the difference between the number of spin-up and spin-down electrons (i.e. the number of unpaired electrons) on the Fe atom. To determine the number of electrons attributed to Fe, we use the Mulliken population analysis.[10] Within our static DFT calculations, we have also checked a different method for determining atomic charges, DDEC6.[11,12] The trends described in the main text are maintained, although the absolute values of the Fe charge are larger for DDEC6 than for the Mulliken population analysis.

Supplementary Note 1: Spin-polarized Projected Densities of States

We extract Spin-polarized Projected Densities of States (pDOS) using the available implementation in CP2K. The electronic density, separated between the spin-up and spin-down channels, is projected onto the $d_{\pm 2}$, $d_{\pm 1}$ and d_0 orbitals. The d_0 corresponds to the d_{z^2}, and by comparison with known ligand field theory we attribute d_{+2} and d_{-2} to d_{xy} and $d_{x^2-y^2}$, respectively. However, the $d_{\pm 1}$ are a combination of the usual d_{xz} and d_{yz} orbitals, so that we denote them by $d_{(xz)yz}$ in the main text. Supplementary Figure 3 informs about the number and nature of magnetic d-orbitals (containing one unpaired electrons in the spin-up channel). To distinguish better between the spin-up and spin-down chanels, we plot the negative of the pDOS for the spin-down chanel (pDOS < 0). The magnetic orbitals are those for which the spin-up pDOS peaks lie below the Fermi level ($E = 0$), while the spin-down pDOS peaks are shifted above the Fermi level. As a result the molecular Fe-porphyrin system shown in panel A and the Fe-porphyrin defect (panel B) possess two magnetic orbitals (d_{+1} and d_{z^2} in energetic order). The Fe-pyridine defect (panel C) also has two magnetic orbitals (d_{z^2} and d_{-1} in energetic order, in contrast with the Fe-porphyrin defect).

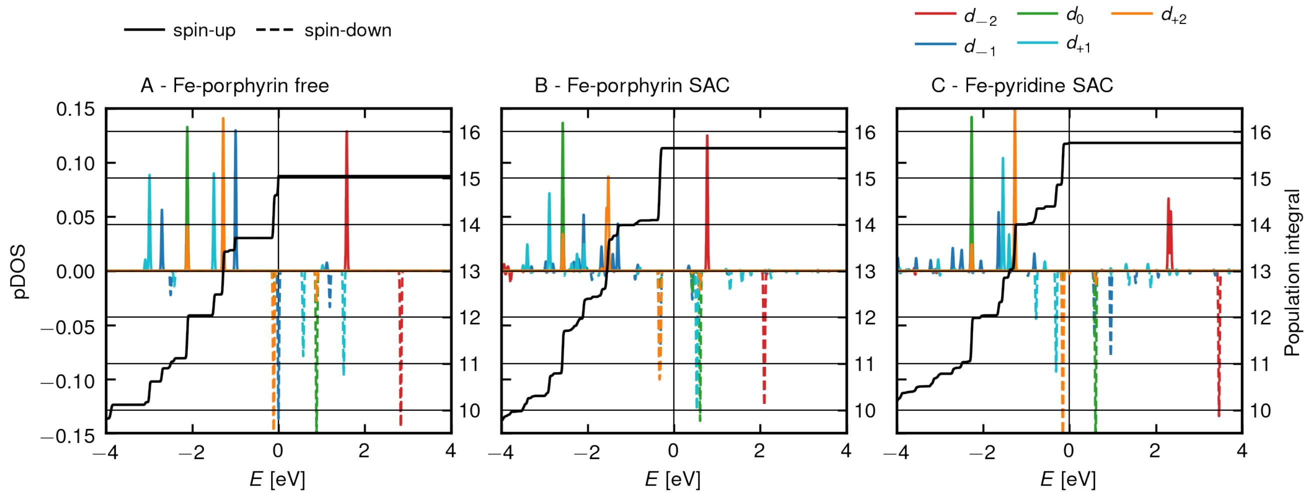

Supplementary Figure 3: Spin-Polarized Projected Density of States (pDOS) onto the d-shell of the Fe center for a free Fe-porphyrin (A - not embedded in graphene, for the Fe-porphyrin SAC defect (B) and for the Fe-pyridine SAC defect (C). The energy is given with respect to the Fermi level. To distinguish better between the spin-up and spin-down chanels, we plot the negative of the pDOS for the spin-down chanel (pDOS < 0). The non-symmetry of the spin-up and spin-down chanels population is a fingerprint of the magnetism of the system.

Supplementary Note 2: DFT-MD simulations at different multiplicities

We conduct DFT-MD simulations of solvated Fe-SACs at different spin settings. Supplementary Table 1 and Supplementary Figure 4 report simulations details and average values and distributions of different properties analysed in this work. Only quantities with $M = 1$ are shown in the main text.

Supplementary Table 1: Total running time, average total energy of the hydrated graphene Fe-defects, Fe spin polarization s_{Fe} and Fe charges q_{Fe}, for the different sets of MD simulations. If two values are indicated, the distribution has two peaks, as can be seen in Supplementary Figure 4.

	Spin state	Fe-pyridine				Fe-porphyrin			
		Time [ps]	$\langle E \rangle$ [kcal/mol]	$\langle s_{Fe} \rangle$	$\langle q_{Fe} \rangle [e]$	Time [ps]	$\langle E \rangle$ [kcal/mol]	$\langle s_{Fe} \rangle$	$\langle q_{Fe} \rangle [e]$
$H_2{}^*O$	RKS	-	-	-	-	14.5	-2822563	0	0.42
	UKS $M = 1$	17.3	-2837049	0.0	0.16	20.8	-2822559	0.4	0.44
	UKS $M = 3$	11.3	-2837060	0.2	0.17	11.9	-2822562	2.5	0.57
$^*OH^-$	UKS $M = 1$	11.4	-2836988	0.05	0.17	18.1	-2822612	0.8	0.46
	UKS $M = 3$	12.8	-2836976	0.9 & 2.1	0.27	11.8	-2822633	1.0	0.48
E-field	RKS	-	-	-	-	39.3	-	0	0.43
	UKS $M = 1$	16.9	-	0.0	0.17	20.5 + 16.9	-	0.5 & 0.9	0.45
	UKS $M = 3$	10.3	-	2.0	0.28	12.6	-	2.5 & 2.6	0.56

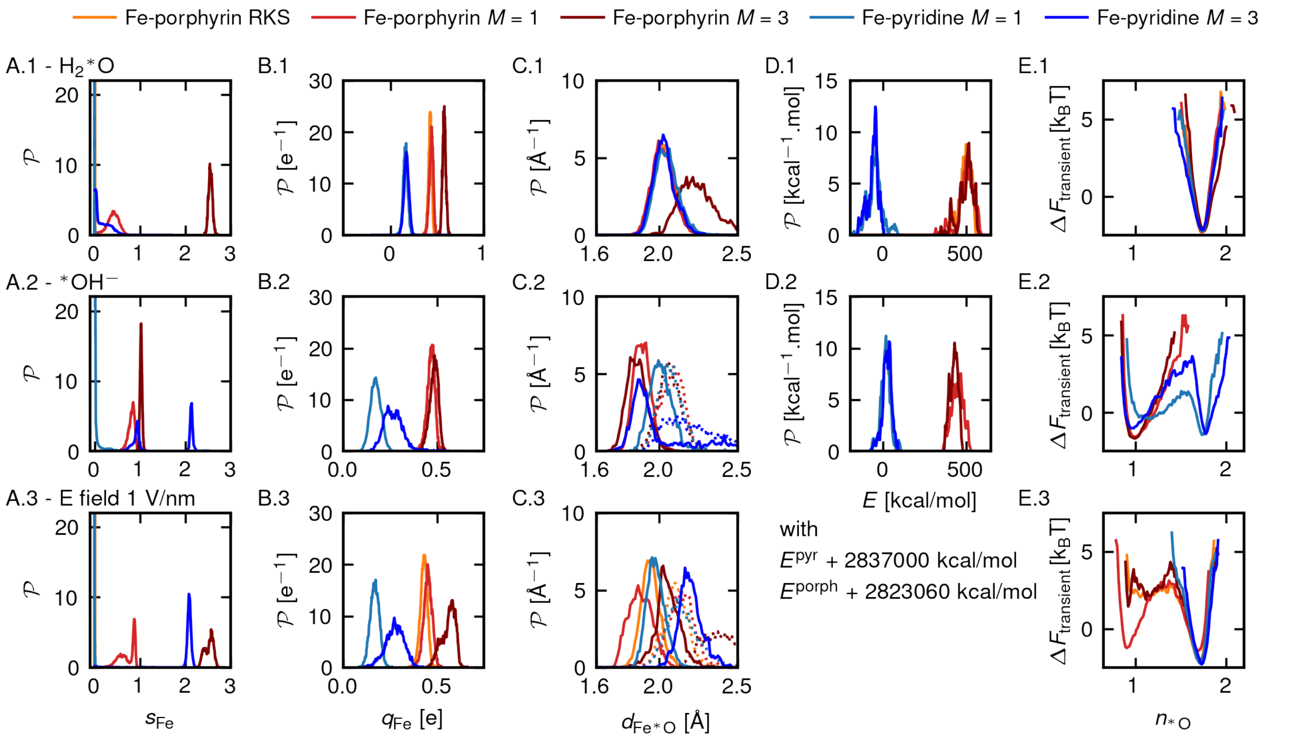

Supplementary Figure 4: Distributions of the Fe spin polarization s_{Fe} (A), Fe charge q_{Fe} (B), the Fe-*O distance (C), the total energy (D) and the "transient" free energy profile of the coordination number (E) for simulations with two adsorbed water molecules (.1), with initially a single hydroxide ion adsorbed at the catalytic site (.2) or with an applied electric field and no hydroxide ion in the initial state (.3), for different sets of MD simulations described in the legend and in Supplementary Table 1. For the total energy distributions, we shift them by a constant value depending on the defect for better visualisation.

Supplementary Note 3: Comparison with a hybrid exchange-correlation functional

We also perform single point energy calculations with a hybrid exchange correlation functional, using PBE0-D3 with dispersion correction.[7,13] We treat the hartree fock exchange (HFX) with the auxiliary density matrix method[14] using FIT3 and FIT11 auxiliary basis sets. The HFX coulomb potential is truncated at a radius of 6 Å. All other parameters (basis set, pseudopotential, cutoff) are identical to the earlier PBE calculations.

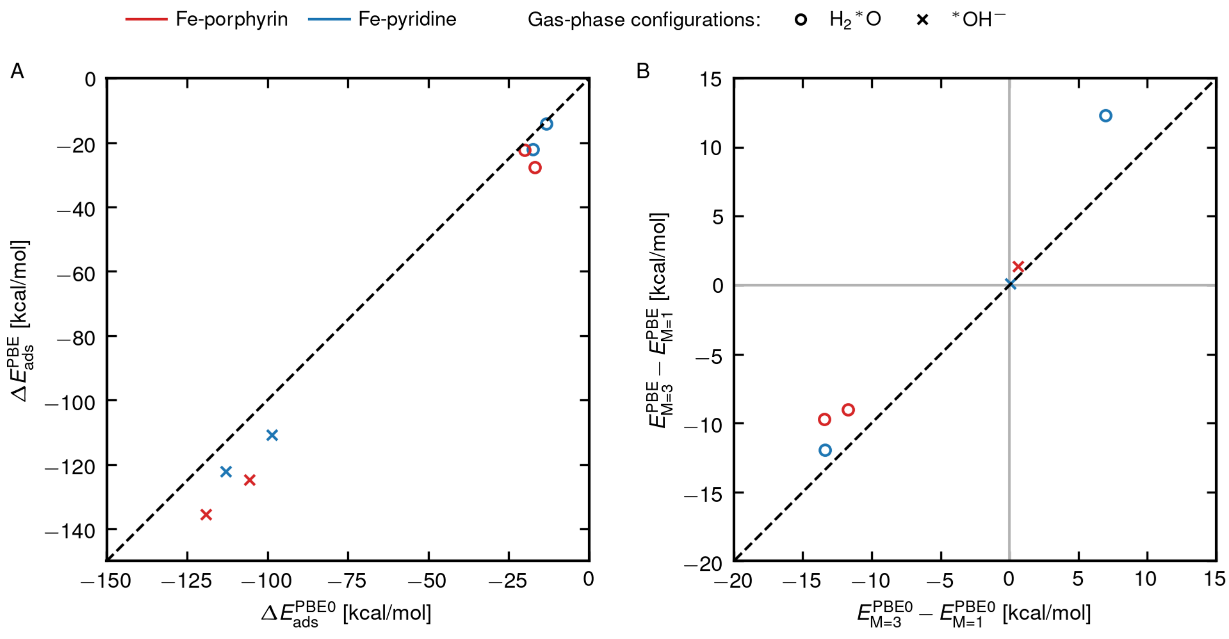

Supplementary Figure 5: Comparison of PBE (y-axis) with PBE0 (x-axis), for a selection of gas-phase configurations (presented in Table II of the main text) for the Fe-pyridine defect (blue circles) and the Fe-porphyrin defect (red circles). (A) BSSE-corrected adsorption energies of either two water molecules or a hydroxide ion and a water molecule, obtained from single point calculations. (B) Energy differences between system multiplicities $M = 3$ and $M = 1$, evaluated from two single point calculations of the same configuration.

We first study the adsorption energy of either two water molecules or of a hydroxide ion on the Fe-defects comparing PBE and PBE0, using the geometry-optimized configurations presented in Table II of the main text. The comparison is shown in Supplementary Figure 5A: for most gas phase configurations with water molecules, the open circles lie close to x=y curve i.e. the absolute difference between PBE and PBE0 results is within 2.5 kcal/mol – as was also observed earlier for H_2O adsorption on SACs.[15] For hydroxide adsorption energies, the crosses deviate below the x=y curve, i.e. the difference is larger, with energies $\sim$ 10 kcal/mol more negative for PBE than PBE0. We note that these calculations are difficult to perform due to different local minima corresponding to different spin states, which render the calculations sensible to the choice of initial conditions. The stability of OH^- on the defects in solution might therefore be overestimated using the classical PBE functional, but the energy difference between PBE and PBE0 results remains roughly constant, *i.e.* the differences observed between

Fe-pyridine and Fe-porphyrin are likely to be robust.

Using the same configurations, we also evaluate the difference in energy between system multiplicities $M = 1$ and $M = 3$, $\Delta E = E_{M3} - E_{M1}$, for PBE and PBE0, by running two single point energy calculations with different system multiplicity. The results are shown in Supplementary Figure 5B. We find that all points lie close to the diagonal, i.e. the energy differences between system multiplicity $M = 1$ and $M = 3$ agree rather well between PBE and PBE0, indicating that the use of PBE is also suitable for our case.

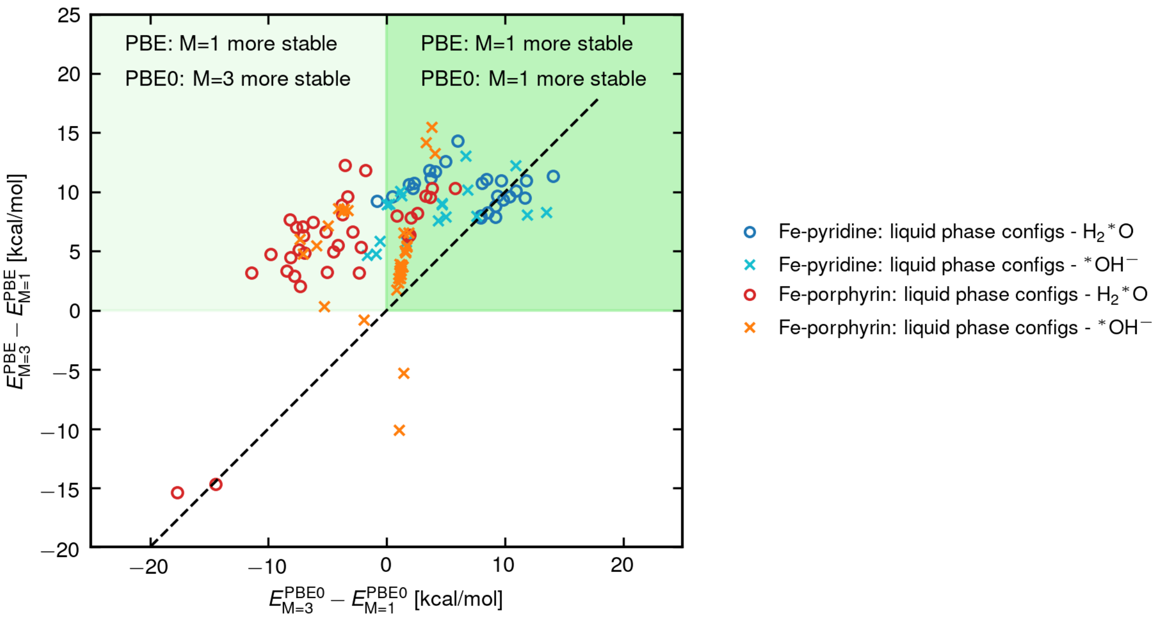

Supplementary Figure 6: Comparison of the energy difference between system multiplicities $M = 3$ and $M = 1$ obtained from PBE with respect to PBE0, for a selection of liquid-phase configurations taken along the DFT-MD trajectories (see Table III in the main text). We distinguish configurations including a hydroxide ion (crosses) or only water molecules (circles), for the Fe-pyridine defect (blue/cyan symbols) and the Fe-porphyrin defect (red/orange symbols). The data lies mostly in the y>0 region (shaded area), where PBE predicts the $M = 1$ multiplicity to be more stable than $M = 3$. The darker green area corresponds to the region where also PBE0 predicts $M = 1$ to be more stable.

To further assess the use of PBE in the liquid phase, we additionally computed ΔE for a set of configurations along the MD simulations with either only liquid water ($H_2{}^*O$) or with an additional hydroxide anion adsorbed on the Fe-defect ($^*OH^-$) and a hydronium cation in the bulk to compensate the charge. The results are shown in Supplementary Figure 6. As expected, the data lies mostly on the left of the $x = y$ curve, *i.e.* PBE0 favors the higher spin state with respect to PBE. For the vast majority of the configurations, PBE predicts that $M = 1$ configurations are energetically more stable than $M = 3$ configurations, which is why we chose to discuss simulations with system multiplicity $M = 1$ in the main text. For Fe-pyridine configurations, PBE0 predicts that the system multiplicity $M = 1$ is lower in energy for most configurations studied. For Fe-porphyrin, although we do observe that there is a fraction of the configurations – mostly with liquid water – for which PBE0 predicts that $M = 3$ is more stable, we

find a significant fraction of configurations for which $M = 1$ is more stable than $M = 3$, especially for configurations with a hydroxide ion.

Since for the PBE functional the $M = 1$ is globally energetically more stable, we present results from simulations with system multiplicity $M = 1$ in the main text for both defect types. For Fe-pyridine systems this is fully consistent with PBE0 results. In case of the Fe-porphyrin defect, PBE0 predicts a system multiplicity of $M = 3$ to be favorable for liquid water while a multiplicity of $M = 1$ is favorable in case of an adsorbed hydroxide ion immersed in water. This indicates that the spin states involved in the prediction of the main result of our work, namely the different stabilization of $^*OH^-$ on the Fe-porphyrin and Fe-pyridine defects, are robust with respect to the choice of functional.

Supplementary Note 4: Trajectories with an initial hydroxide $^*OH^-$

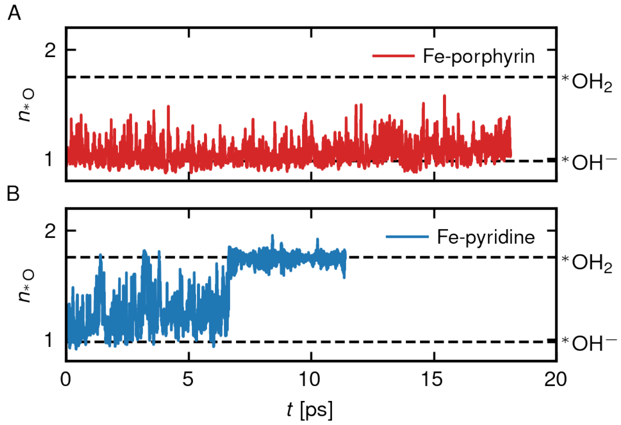

Supplementary Figure 7: Coordination number $n_{*\mathrm{O}}$ as a function of time for the simulations ($M = 1$) starting with an hydroxide ion adsorbed to the Fe-SAC catalytic site, for Fe-porphyrin (A) and for Fe-pyridine (B). In the latter, the hydroxide ion recombines with the hydronium after 6 ps.

Supplementary Note 5: Electric field

We apply a finite field as implemented in the software CP2K, following the method introduced by Umari and Pasquerello[16] which accounts for the Berry phase. The so-applied constant force density corresponds to a displacement field of nominally $D_0/\varepsilon_0 = 1$ V/nm. To estimate the local electric field induced by the applied displacement field, we benchmark the effect of an E-field on bulk water. For this, we perform MD simulations of a 20.73 x 20.73 x 20.73 Å^3 box containing 300 water molecules, both at the force field level, using the SPC/E water model,[1] and at the DFT level, using the BLYP functional[17,18] with Grimme's D3 dispersion correction[7] and the DZVP-MOLOPT-SR basis set,[8] combined with GTH pseudopotentials.[9] In these bulk simulations, the externally applied field corresponds to the average electric field in the liquid. Supplementary Figure 8A shows the average orientation of water molecules $\langle\cos\theta\rangle$, with θ the angle between the dipole moment of a water molecule and the direction of the field, as a function of time, for a range of applied field strengths. Supplementary Figure 8B reports the equilibrium values as a function of the field strength and, comparing the mean orientation measured inside the bulk phase of our Fe-SAC systems (horizontal dashed-dotted line), we deduce that the applied force density of $D_0/\varepsilon_0 = 1$ V/nm corresponds to an effective electric field strength of $E_{\text{bulk}} \sim 0.12 \pm 0.02$ V/nm (vertical line), which is small enough to avoid the disruption of the hydrogen bond network in the bulk.

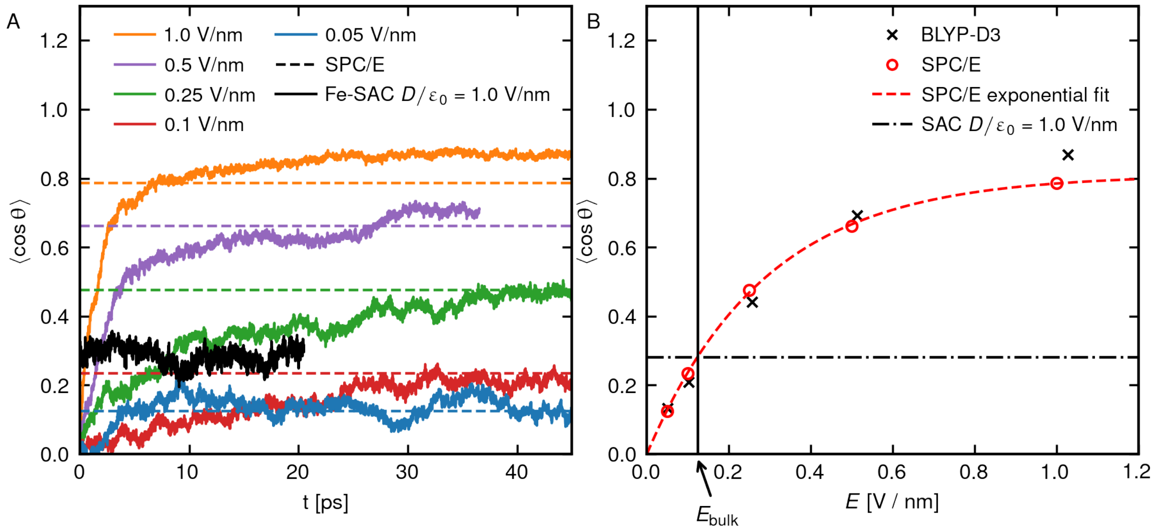

Supplementary Figure 8: (A) Bulk water orientation under an external electric field E, for different field strengths denoted by different colors, as a function of time. Solid lines are results from DFT simulations while the horizontal dashed lines are averages over long force-field MD simulations. The black solid line corresponds to the average orientation of water molecules far from the graphene sheet in the Fe-porphyrin simulation with applied field $D/\varepsilon_0 =$ 1.0 V/nm. (B) Average bulk water orientation under an external electric field, as a function of the field strength, for DFT-MD simulations (black crosses) and SPC/E force-field MD simulations (red circles). The red dashed line is an exponential fit to the SPC/E water data used for our benchmark. The horizontal dashed line corresponds to the Fe-porphyrin system and the vertical line indicates the obtained field strength for the Fe-porphyrin system.

Supplementary References

(1) Berendsen, H. J. C.; Grigera, J. R.; Straatsma, T. P. The missing term in effective pair potentials. *The Journal of Physical Chemistry* **1987**, *91*, 6269–6271.

(2) Oostenbrink, C.; Villa, A.; Mark, A. E.; Van Gunsteren, W. F. A biomolecular force field based on the free enthalpy of hydration and solvation: The GROMOS force-field parameter sets 53A5 and 53A6. *Journal of Computational Chemistry* **2004**, *25*, 1656–1676.

(3) Abraham, M. J.; Murtola, T.; Schulz, R.; Páll, S.; Smith, J. C.; Hess, B.; Lindahl, E. GROMACS: High Performance Molecular Simulations through Multi-Level Parallelism from Laptops to Supercomputers. *SoftwareX* **2015**,

(4) Kühne, T. D. et al. CP2K: An electronic structure and molecular dynamics software package - Quickstep: Efficient and accurate electronic structure calculations. *The Journal of Chemical Physics* **2020**, *152*, 194103.

(5) Perdew, J. P.; Burke, K.; Ernzerhof, M. Generalized Gradient Approximation Made Simple. *Physical Review Letters* **1996**, *77*, 3865–3868.

(6) Perdew, J. P.; Burke, K.; Ernzerhof, M. Generalized Gradient Approximation Made Simple [Phys. Rev. Lett. 77, 3865 (1996)]. *Physical Review Letters* **1997**, *78*, 1396–1396.

(7) Grimme, S.; Antony, J.; Ehrlich, S.; Krieg, H. A consistent and accurate ab initio parametrization of density functional dispersion correction (DFT-D) for the 94 elements H-Pu. *The Journal of chemical physics* **2010**, *132*, 154104.

(8) VandeVondele, J.; Hutter, J. Gaussian basis sets for accurate calculations on molecular systems in gas and condensed phases. *The Journal of Chemical Physics* **2007**, *127*.

(9) Goedecker, S.; Teter, M.; Hutter, J. Separable dual-space Gaussian pseudopotentials. *Phys. Rev. B* **1996**, *54*, 1703–1710.

(10) Mulliken, R. S. Electronic Population Analysis on LCAO–MO Molecular Wave Functions. I. *The Journal of Chemical Physics* **2004**, *23*, 1833–1840.

(11) Limas, N. G.; Manz, T. A. Introducing DDEC6 atomic population analysis: part 2. Computed results for a wide range of periodic and nonperiodic materials. *RSC Advances* **2016**, *6*, 45727–45747.

(12) Manz, T. A.; Limas, N. G. Introducing DDEC6 atomic population analysis: part 1. Charge partitioning theory and methodology. *RSC Advances* **2016**, *6*, 47771–47801.

(13) Adamo, C.; Barone, V. Toward reliable density functional methods without adjustable parameters: The PBE0 model. *The Journal of Chemical Physics* **1999**, *110*, 6158–6170.

(14) Guidon, M.; Hutter, J.; VandeVondele, J. Auxiliary density matrix methods for Hartree- Fock exchange calculations. *Journal of chemical theory and computation* **2010**, *6*, 2348–2364.

(15) Patel, A. M.; Ringe, S.; Siahrostami, S.; Bajdich, M.; Nørskov, J. K.; Kulkarni, A. R. Theoretical Approaches to Describing the Oxygen Reduction Reaction Activity of Single-Atom Catalysts. *The Journal of Physical Chemistry C* **2018**, *122*, 29307–29318.

(16) Umari, P.; Pasquarello, A. Ab initio Molecular Dynamics in a Finite Homogeneous Electric Field. *Physical Review Letters* **2002**, *89*, 157602.

(17) Becke, A. D. Density-functional exchange-energy approximation with correct asymptotic behavior. *Phys. Rev. A* **1988**, *38*, 3098–3100.

(18) Lee, C.; Yang, W.; Parr, R. G. Development of the Colle-Salvetti correlation-energy formula into a functional of the electron density. *Phys. Rev. B* **1988**, *37*, 785–789.
