# Supplementary material for: Enhanced interfacial water dissociation on a hydrated iron porphyrin single-atom catalyst in graphene
Source: Commun Chem. 2023 Nov 2;6:236. doi: 10.1038/s42004-023-01027-9 (PMC10622426; doi:10.1038/s42004-023-01027-9)
Supplement: Supplementary file 3 — Description of Additional Supplementary Files [file 42004_2023_1027_MOESM3_ESM.pdf]

Description of Additional Supplementary Files

File name: Supplementary Movie 1

Description: Movie of a Fe-pyridine defect in water, where the two adsorbed water molecules are highlighted.

File name: Supplementary Movie 2

Description: Movie of a Fe-porphyrin defect in water, where the two adsorbed water molecules are highlighted.

File name: Supplementary Movie 3

Description: Movie of a 1.1 ps excerpt of a simulation of Fe-pyridine (corresponding to the trajectory in Supplementary Figure 7b) started with a hydroxide ion adsorbed on the catalytic site, during which we observe the recombination of $^{*}OH^{-}$ with H_3O^{+}

File name: Supplementary Movie 4

Description: Movie of a 2.8 ps excerpt of a simulation of Fe-porphyrin in the presence of an electric field perpendicular to the graphene sheet (from left to right in the video), during which a proton transfer is observed. Fig. 4f-h of the main text were obtained from the same trajectory.
